# Effects of *Lactobacillus plantarum* and *Pediococcus acidilactici* co-fermented feed on growth performance and gut microbiota of nursery pigs

**DOI:** 10.3389/fvets.2022.1076906

**Published:** 2022-12-12

**Authors:** Yuzeng Yang, Guohua Yan, Xianhua Meng, Xu Wang, Zhiqiang Zhao, Shuguang Zhou, Guangdong Li, Qiuliang Zhang, Xiaoyuan Wei

**Affiliations:** ^1^Institute of Animal Husbandry and Veterinary Medicine of Hebei Province, Baoding, China; ^2^Hebei Provincial Animal Husbandry Station, Shijiazhuang, China; ^3^Institute of Agro-Resources and Environment, Hebei Fertilizer Technology Innovation Center, Hebei Academy of Agriculture and Forestry Sciences, Shijiazhuang, China; ^4^Baoding Animal Husbandry Workstation, Baoding, China; ^5^Division of Agriculture, Department of Animal Science, University of Arkansas, Fayetteville, AR, United States

**Keywords:** fermented feed, *Lactobacillus plantarum*, *Pediococcus acidilactici*, nursery pig, growth performance, gut microbiota

## Abstract

The fermented feed has been used extensively as a growth promoter in agricultural animal production. However, the effects of fermented feed on swine gut microbiota are still largely unknown. The work presented here aimed to investigate the growth performance and gut microbiota of nursery pigs receiving the LPF diet (10% *Lactobacillus plantarum* and *Pediococcus acidilactici* co-fermented feed + basal diet) compared with pigs receiving the NC diet (basal diet). The data showed LPF diet numerically improved average daily gain and significantly increased fecal acetate, butyrate, and total short-chain fatty acid (SCFA) concentrations. Furthermore, gut microbiota structure and membership significantly changed in response to the addition of fermented feed in the diet. Gut microbiota results indicated that LPF treatment significantly enriched SCFA-producing bacteria such as *Megasphaera, Roseburia, Faecalibacterium, Blautia, Selenomonas, Dialister, Acidaminococcus, Ruminococcus*, and *Bifidobacterium*. Some of these bacteria also had anti-inflammatory and other beneficial functions. Overall, these findings suggested that *Lactobacillus plantarum* and *Pediococcus acidilactici* co-fermented feed benefited growth performance and established potential health impacts on the gut microbiota of nursery pigs.

## Introduction

Nowadays the beneficial effects of fermented feed on animal growth performance and health have received increasing attention. Fermentation is a metabolic process that produces biochemical changes in the primary food matrix through the action of microorganism enzymes (1). The importance of fermented feed is to improve the nutrient digestibility of raw material (e.g., corn and soybean meal) and increase the availability of vitamins and minerals by reducing the level of anti-nutritional factors (2). Apart from improved nutritional quality, fermented feed could benefit animal growth performance by boosting immune function and improving intestinal morphology (3, 4). Zhou et al. reported that fermented feed not only increased serum immunoglobulin levels but also improved lymphocyte proliferation and transformation (5). Another study revealed that supplementing piglet diets with fermented soybean meal significantly increased both villus height and villus: crypt ratio, which enlarged intestinal surface area and thereby improved nutrition uptake ability (6). Furthermore, Kiers et al. determined that fermented soybeans promoted feed intake and weight gain, as well as reduced diarrhea incidence of weaned piglets challenged with *Escherichia coli* (7).

Evidence is accumulating to demonstrate that incorporating fermented feed into swine diets has many beneficial effects on intestinal microbiota. Lactic acid-producing bacteria such as *Lactobacillus plantarum* and *Pediococcus acidilactici* are commonly used for fermentation, which generates a large amount of lactic acid and lowers the pH of the feed. Thus, the fermented feed can inhibit pathogenic bacterial growth, deliver probiotics, and prevent pathogenic bacteria from attaching to the intestinal walls (8). Studies have shown that fermented products reduced the proliferation of certain enteropathogens like *E. coli* and *Salmonella* in both swine and broiler (4, 8).

Although previous studies have remarkably expanded our knowledge regarding the impacts of fermented feed on gut microorganisms, they delivered information only on a limited set of microbial taxa. A gap in the understanding of how fermented feeds modulate the entire complex gut microbial ecosystem still exists. In this study, we tested *Lactobacillus plantarum* and *Pediococcus acidilactici* co-fermented feed and applied next-generation sequencing technology to achieve a depth insight into how the gut microbiota of nursery pigs evolves under the influence of fermented feed.

## Materials and methods

Animal management and care followed the Institute of Animal Husbandry and Veterinary Medicine of Hebei Province Animal Care and Use Committee guidelines (IAHVM20190910-1, Hebei, China).

### Animals and experimental design

This study was carried out at a commercial swine farm (Zhangjiakou, China). On the weaning day, a total of 32 piglets (body weight 14.65 ± 0.46 kg; 25 ± 1 d of age) were transferred to a nursery facility and were randomly assigned to the negative control (NC, *n* = 16) or fermented feed (LPF, *n* = 16) groups. The ambient temperature was set at 30°C upon pig arrival and was reduced 2°C per week until a 24°C setting was achieved. Each pen was fully slatted (1.8 × 2.0 m^2^) and was equipped with a nipple drinker and a feeder for *ad libitum* access to diets and water. Each pen housed eight pigs. After a 14-day adaption period, a 31-day feeding trial was conducted. All pigs were supplied with a common diet during the adaption period and then switched to the experimental diets at 39-day old. NC diet: basal diet; LPF diet: NC + 10% *Lactobacillus plantarum* and *Pediococcus acidilactici* co-fermented feed (M108, Dacheng (Wanda) Tianjin Co., Ltd, China). Diets meet or exceed the NRC (9) nutrient requirements and the Supplementary Tables S1, S2 detailed the basal diet and LPF diet formulations.

### Data recording and sample collection

Individual pig body weight (BW) was measured at the beginning and the end of the study. Each dietary treatment data was used to calculate average daily gain (ADG).

Blood samples (*n* = 5) were collected into tubes *via* jugular vena cava from randomly selected pigs (69-day-old) at the end of the study and were assayed for total protein (TP), blood urea nitrogen (BUN), glutamate oxaloacetate transaminase (GOT), glutamate pyruvate transaminase (GPT), and superoxide dismutase (SOD). At the same time, ~1 g (*n* = 4) and 2 g (*n* = 10) rectal swab samples for individual animals were randomly collected for short-chain fatty acid analysis and gut microbiota analysis, respectively. Samples were temporally kept on dry ice and then stored in an ultra-low temperature freezer until further analysis.

### Growth performance data analysis

Independent Samples *T*-Test program in IBM SPSS 22.0 statistical software was used for data statistical analysis and each animal served as the experimental unit. The probability value of *p* < 0.05 was considered significant.

### 16S rRNA sequencing and data analysis

Microbial genome DNA was extracted from the fecal samples using the DNeasy PowerLyzer PowerSoil Kit (Qiagen, Germantown, MD, USA), following the manufacturer's instructions. DNA concentration and purity were decided by NanoDrop One (Thermo Fisher Scientific, Madison, WI, USA) and then diluted to 20 ng/μL for downstream application.

The 16S rRNA gene hypervariable regions V3–V4 were used to identify bacteria and were amplified using primers 341F (5′-ACTCCTACGGGAGGCAGCA-3′) and 806R (5′-GGACTACHVGGGTWTCTAAT-3′). PCR reaction conditions are initial denaturation at 98°C for 2 min, followed by 30 cycles, including denaturation at 98°C for 15 s, annealing at 55°C for 30 s, and extension at 72°C for 30 s, with a final extension at 72°C for 5 min and 4°C hold. Agarose gel (1.2%) electrophoresis was applied to assess the success of PCR reactions. In addition, VAHTS DNA Clean Beads (Vazyme Biotech, Nanjing, Jiangsu, China) and Quant-iT PicoGreen dsDNA Assay Kit (Invitrogen, Carlsbad, CA, USA) were used to purify and quantify the amplicons, respectively. Purified PCR amplicons were then pooled together to generate a sequencing library. Agilent 2100 Bioanalyzer (Agilent, Santa Clara, CA, USA) and the Quant-iT PicoGreen dsDNA Assay Kit were applied to detect the quality and the concentration of the library, respectively. To detect potential bias introduced during PCR amplification and the MiSeq run, a mock community [ZymoBIOMICS™Microbial Community Standard (Zymo, Irvine, CA, USA)] was included in the sequencing library as a standard. Finally, the library was sequenced on the Illumina MiSeq sequencer with MiSeq Reagent Kit V3 (600 cycles) to generate paired-end reads.

The fastq files downloaded from the Illumina sequencer were analyzed using the QIIME2 (2019.4 release) microbiome bioinformatics platform (10). QIIME 2 plugin DADA2 processed sequencing data including quality control, denoising, merging, and removing chimera as well as singleton, and generated a feature table for the downstream analysis (11). Greengenes reference database (V13_8) trained Naive Bayes classifier was used to annotate sequences (12, 13).

Alpha diversity and beta diversity were estimated in QIIME2 at a sub-sampling depth of 62380 sequences for each sample. The analysis of similarity (ANOSIM) was performed to compare the dissimilarity between the treatments. In addition, the most differentially abundant bacteria between treatments were identified by LEfSe (Linear discriminant analysis Effect Size, LDA score > 2) at each taxonomic rank. Random forest R package with default setting was used to identify microbial signatures that best differentiate treatments at the feature level (14).

## Results

### The effects of co-fermented feed on swine growth performance

**Figure 1:**
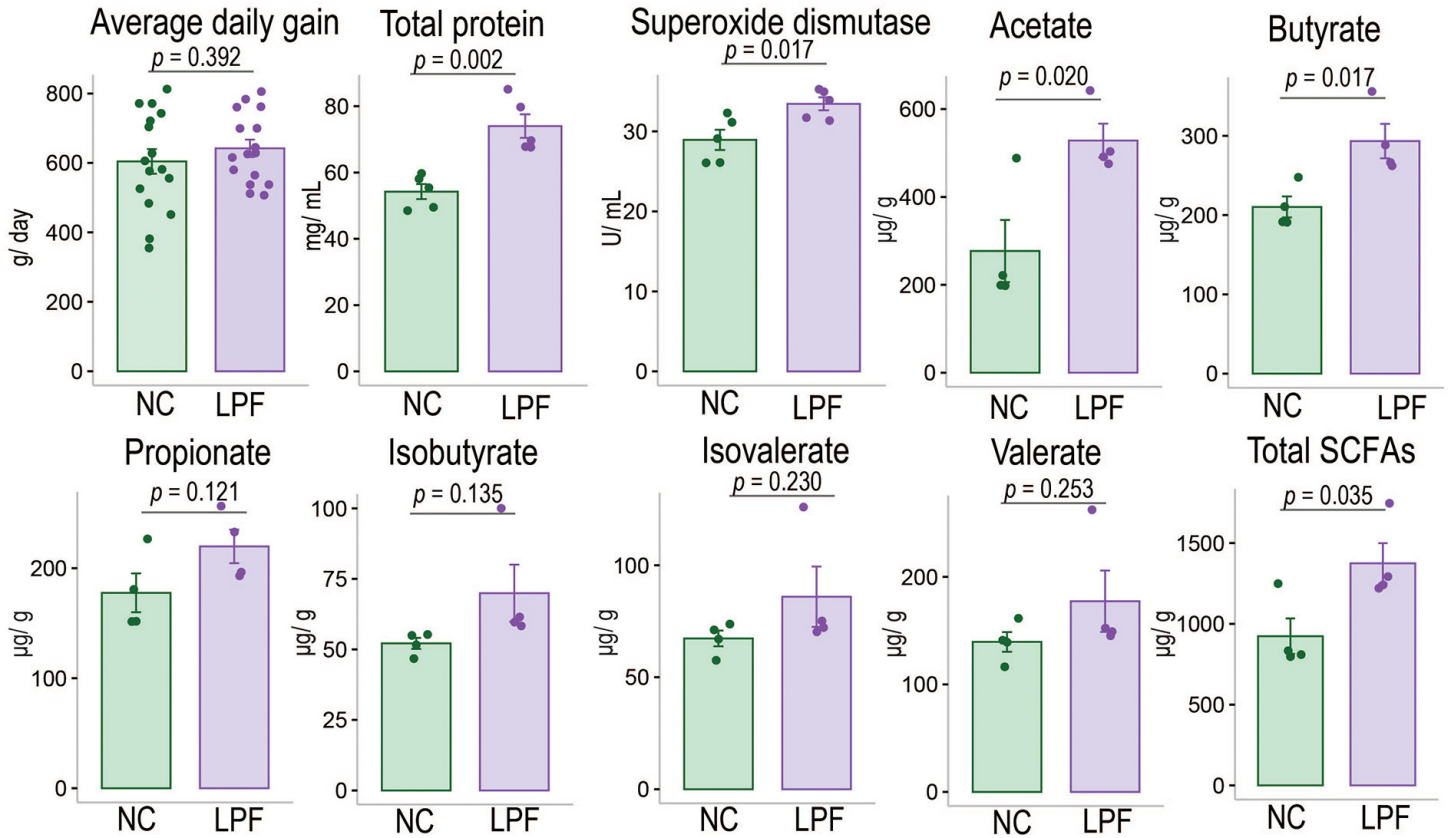
Effects of LPF diet on average daily gain, serum indexes, and fecal SCFAs of nursery pigs compared with NC diet. NC diet: basal diet, LPF diet: NC + 10% *Lactobacillus plantarum* and *Pediococcus acidilactici* co-fermented feed.

Experimental data such as BW was provided in Supplementary Table S3. The results indicated that the LPF diet numerically improved average daily gain (*p* = 0.392) and significantly improved the TP concentration (*p* = 0.002) as well as SOD (*p* = 0.017) compared with the NC diet (Figure 1). We also found that the LPF diet had no significant impacts on BUN, GPT, and GOT (Supplementary Table S4).

As shown in Figure 1, the LPF diet numerically improved the concentrations of propionate, isobutyrate, isovalerate, as well as valerate (*p* > 0.05) and significantly improved acetate (*p* = 0.020), butyrate (*p* = 0.017), and total SCFAs (*p* = 0.035) compared with NC diet (more detailed information are provided in Supplementary Table S5).

### The influence of co-fermented feed on gut microbial diversity

**Figure 2:**
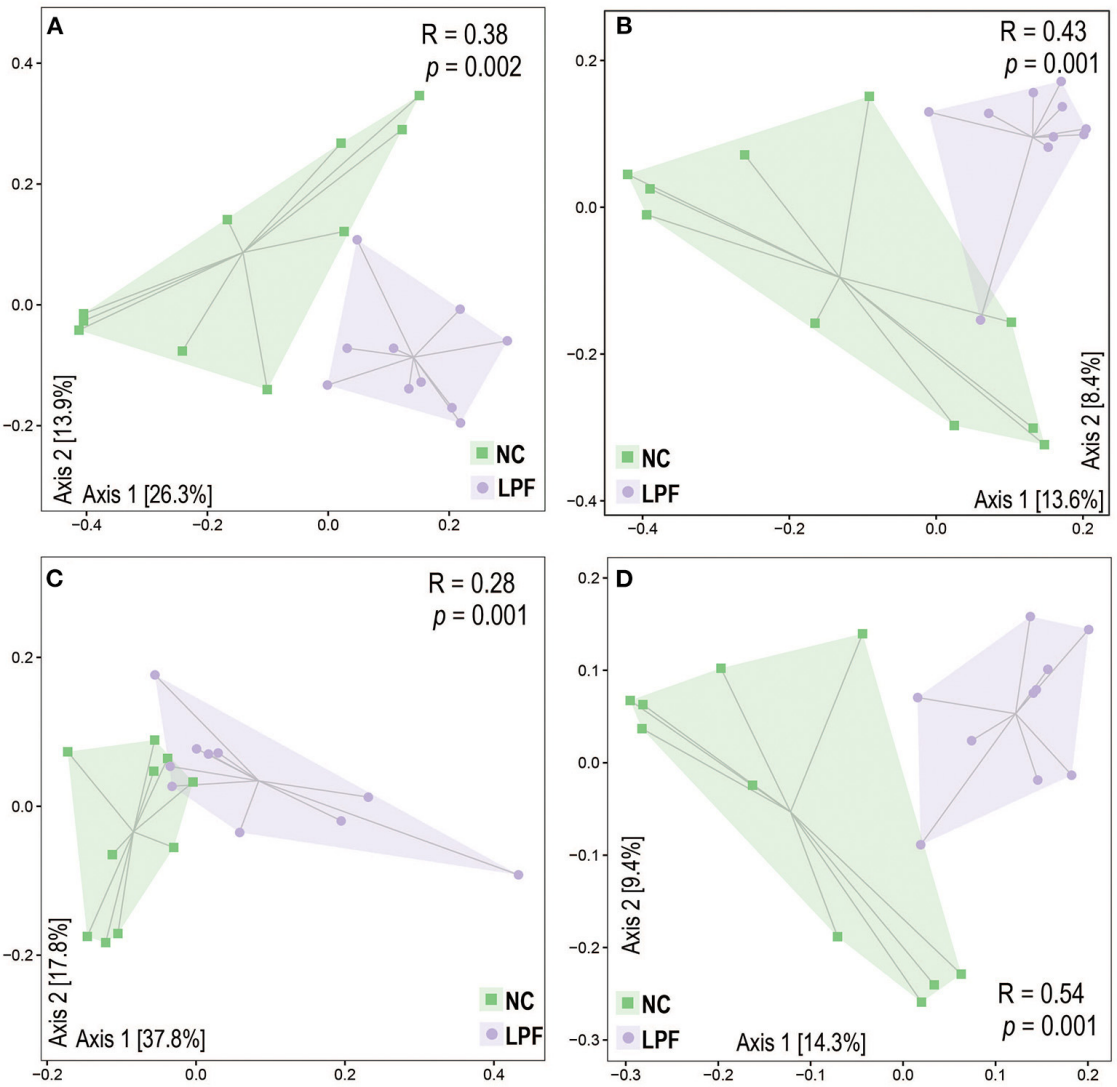
LPF diet modulated gut microbiota beta diversity based on **(A)** Bray-Curtis, **(B)** Jaccard, **(C)** Weighted UniFrac, and **(D)** Unweighted UniFrac distances. The analysis of similarity (ANOSIM) was applied to estimate the dissimilarity between NC and LPF treatments. NC diet: basal diet, LPF diet: NC + 10% *Lactobacillus plantarum* and *Pediococcus acidilactici* co-fermented feed.

The gut microbiota alpha diversity was measured by the Shannon index, Observed_species, and Chao1, however, there were no significant differences between the treatments (Supplementary Figure S1). Beta diversity, including Bray-Curtis, Jaccard, Weighted Unifrac, and Unweighted Unifrac distances, was used to determine gut microbiota structural changes in response to co-fermented feed (Figure 2). The gut microbiota profiles of the NC and LPF groups were distinctly different. Principal coordinate analysis (PCoA) based on Bray-Curtis dissimilarity and Jaccard distance showed remarkable clusters for each experimental group. PCoA based on the Weighted and Unweighted Unifrac distances also revealed the distinct changes induced by co-fermented feed. The analysis of similarities (ANOSIM) confirmed the pattern that the swine gut microbiome was significantly different between NC and LPF treatments (Bray-Curtis: *R* = 0.38, *P* = 0.002; Jaccard: *R* = 0.43, *P* = 0.001; Weighted Unifrac: *R* = 0.28, *P* = 0.001; and Unweighted Unifrac: *R* = 0.54, *P* = 0.001).

### Gut microbiota composition changes induced by co-fermented feed

**Figure 3:**
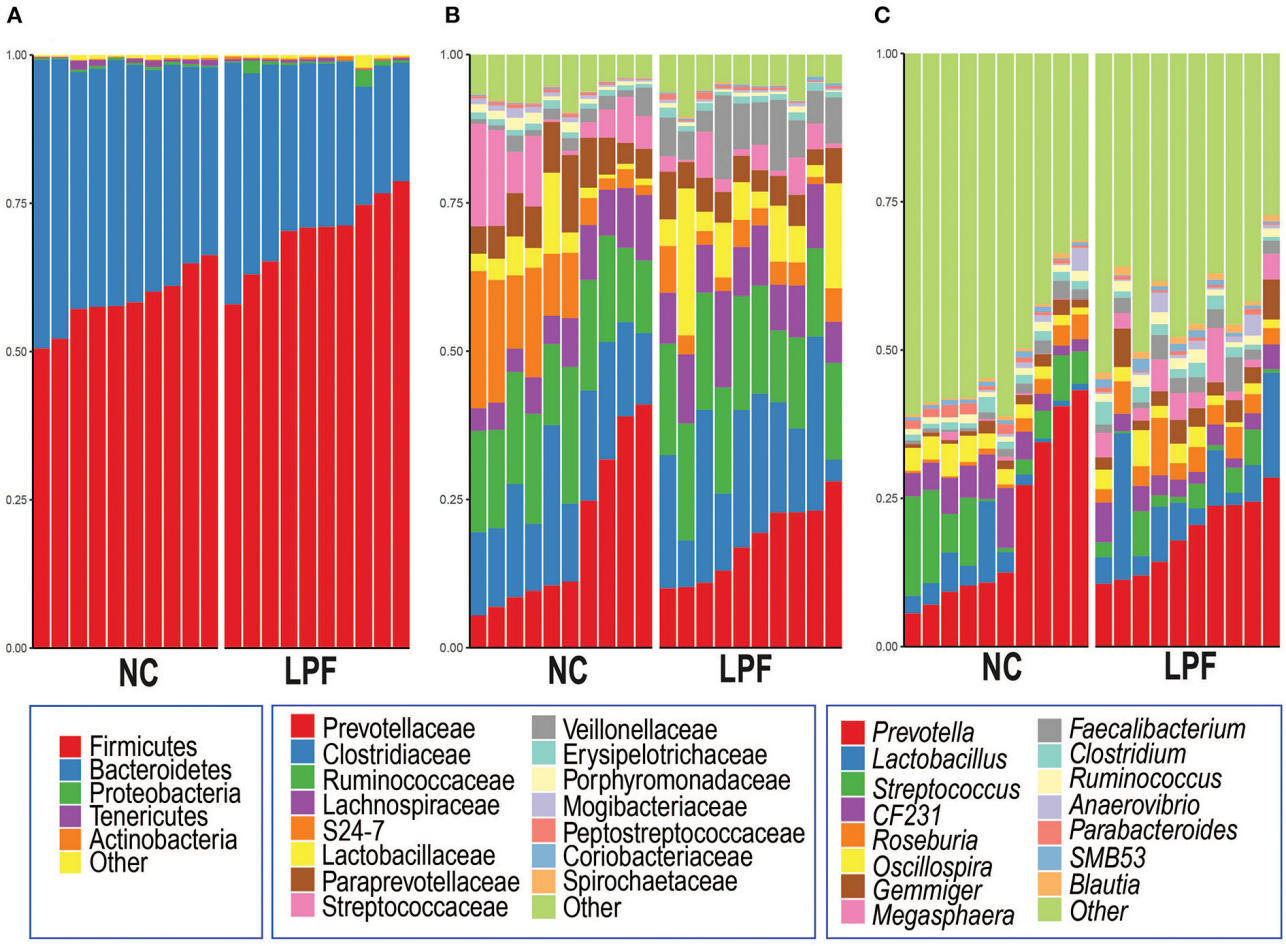
Gut microbiota compositions at **(A)** phylum, **(B)** family, and **(C)** genus levels for each treatment. NC diet: basal diet, LPF diet: NC + 10% *Lactobacillus plantarum* and *Pediococcus acidilactici* co-fermented feed.

At the phylum level, the two treatment groups had a similar pattern that the dominant phyla Firmicutes and Bacteroidetes accounted for more than 95% of total sequences (Figure 3A). The top 15 bacteria at the family level are shown in Figure 3B, the most represented bacteria are Prevotellaceae, Clostridiaceae, and Ruminococcaceae in both groups. But the subdominant gut microbiota component varied at different treatments. For example, S24-7 and Streptococcaceae, the subdominant gut microbiota component in NC (10.7 and 7.4%, respectively) group, strikingly decreased in the LPF (3.8 and 3.1%, respectively) treatment. In addition, the relative abundance of Veillonellaceae was dramatically promoted by the LPF diet compared to that in the NC diet (7.5 vs. 1.9%). At the genus level (Figure 3C), *Prevotella* is the predominant bacteria in both groups (NC: 20.1% and LPF: 18.7%) among the top 15 genera. The relative abundance of *Lactobacillus* (8.6 vs. 3.8%), *Roseburia* (4.2 vs. 1.6%), *Gemmiger* (3.5 vs. 1.4%), *Megasphaera* (3.8 vs. 0.3%), and *Faecalibacterium* (2.7 vs. 1.3%) were largely enriched by the LPF diet compared to the NC diet. However, *Streptococcus* (7.2 vs. 2.9%) dramatically decreased in the LPF treatment compared to the NC group.

**Figure 4:**
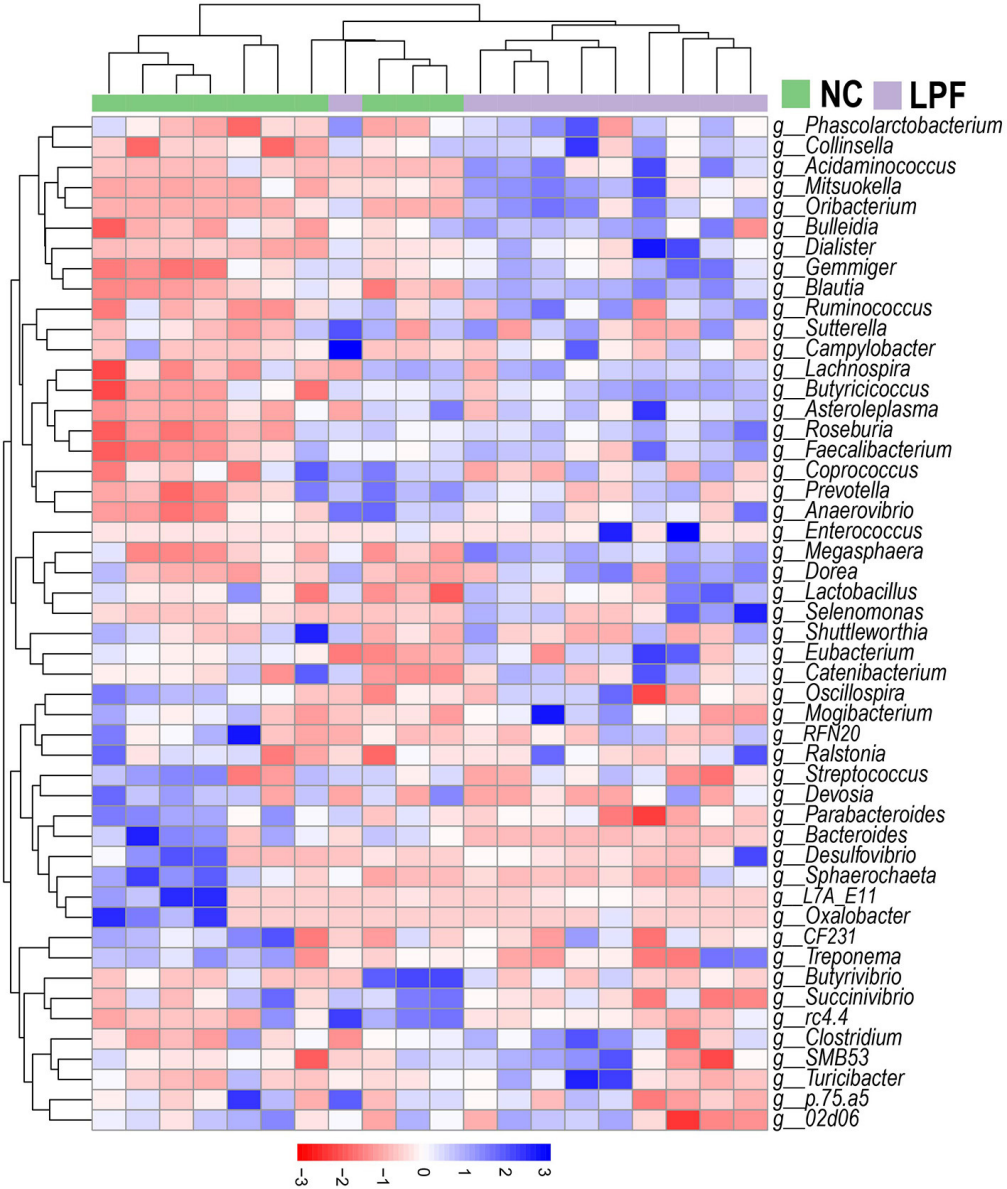
Heatmap of the relative abundance of top 50 genera. Clusters based on Euclidean distance using complete linkage clustering. NC diet: basal diet, LPF diet: NC + 10% *Lactobacillus plantarum* and *Pediococcus acidilactici* co-fermented feed.

A heatmap with cluster analysis showed the abundance of the top 50 bacterial taxa at the genus level, revealing visible compositional differences between piglets that received LPF feed and those fed the NC diet (Figure 4). Complete linkage hierarchical clustering based on Euclidean distance generated a separation of LPF and NC treatments, except for one sample from the LPF group joined the cluster containing samples from the NC group.

### Linear discriminant analysis of the gut microbiota

**Figure 5:**
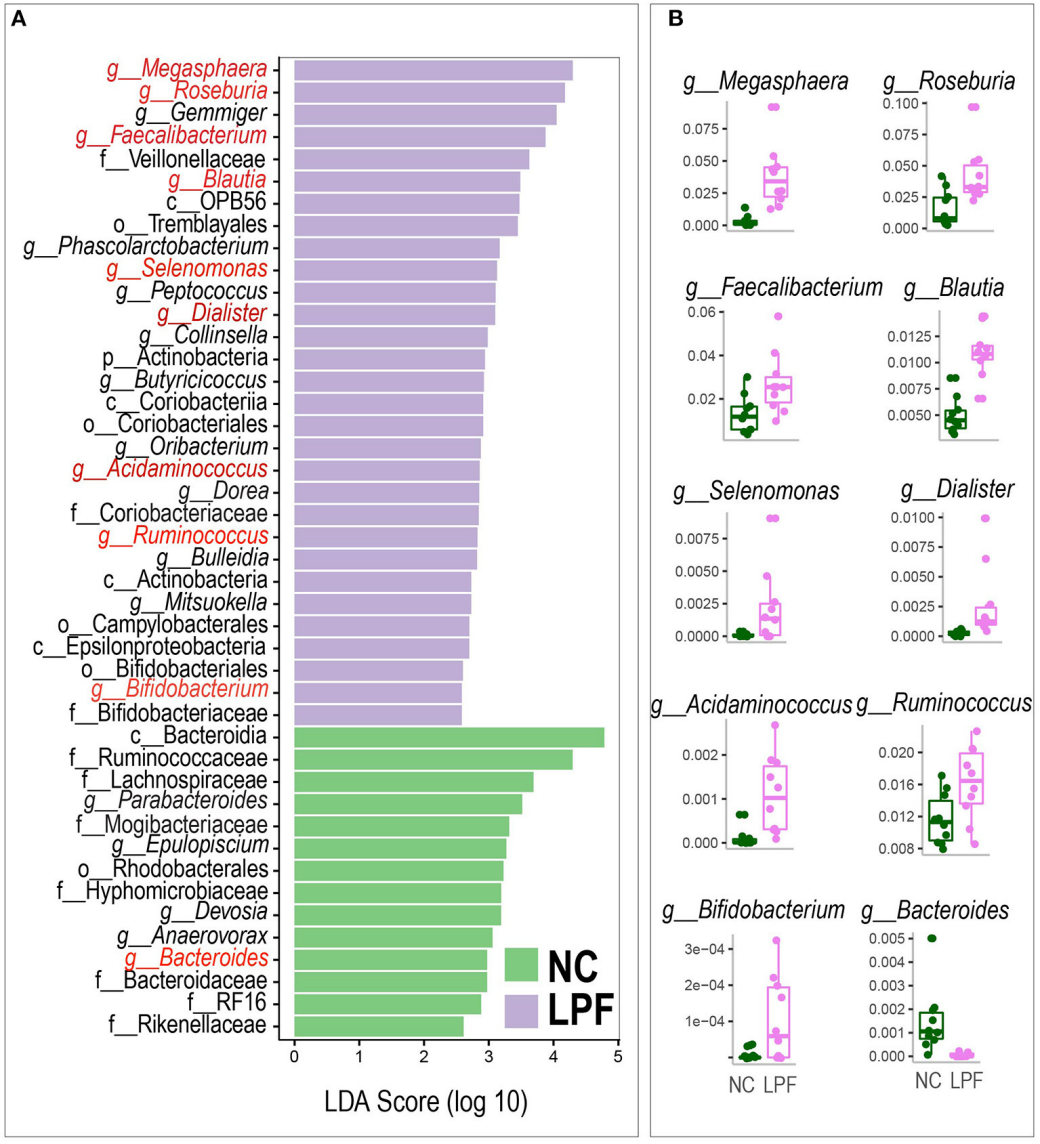
**(A)** Linear discriminant analysis effect size (LefSe) of significantly different relative abundant bacterial taxa between groups. **(B)** Relative abundance of the important bacteria selected by LEfSe. NC diet: basal diet, LPF diet: NC + 10% *Lactobacillus plantarum* and *Pediococcus acidilactici* co-fermented feed.

We next performed LEfSe analysis to detect the most differentially abundant bacterial taxa between the NC and the LPF groups. A total of 44 represented bacterial taxa were identified (Figure 5A). Many biomarker genera like *Megasphaera, Roseburia, Faecalibacterium, Blautia, Selenomonas, Dialister, Acidaminococcus, Ruminococcus*, and *Bifidobacterium* were significantly more abundant in the LPF group compared with the NC group, whereas the relative abundance of *Bacteroides* was distinctly decreased by the LPF diet (Figure 5B).

For the other bacteria such as *Gemmiger, Phascolarctobacterium, Peptococcus, Collinsella, Butyricicoccus, Oribacterium, Dorea, Bulleidia, Mitsuokella*, Veillonellaceae, OPB56, Tremblayales, Actinobacteria, Coriobacteriia, Coriobacteriales, Coriobacteriaceae, Actinobacteria, Campylobacterales, Epsilonproteobacteria, Bifidobacteriales, and Bifidobacteriaceae were also higher in the LFP group, whereas the NC group had significantly more abundant *Epulopiscium, Devosia, Anaerovorax, Parabacteroides*, Bacteroidia, Ruminococcaceae, Lachnospiraceae, Mogibacteriaceae, Rhodobacterales, Hyphomicrobiaceae, Bacteroidaceae, RF16, and Rikenellaceae.

### Gut microbiota signature of pigs fed with co-fermented diet

**Figure 6:**
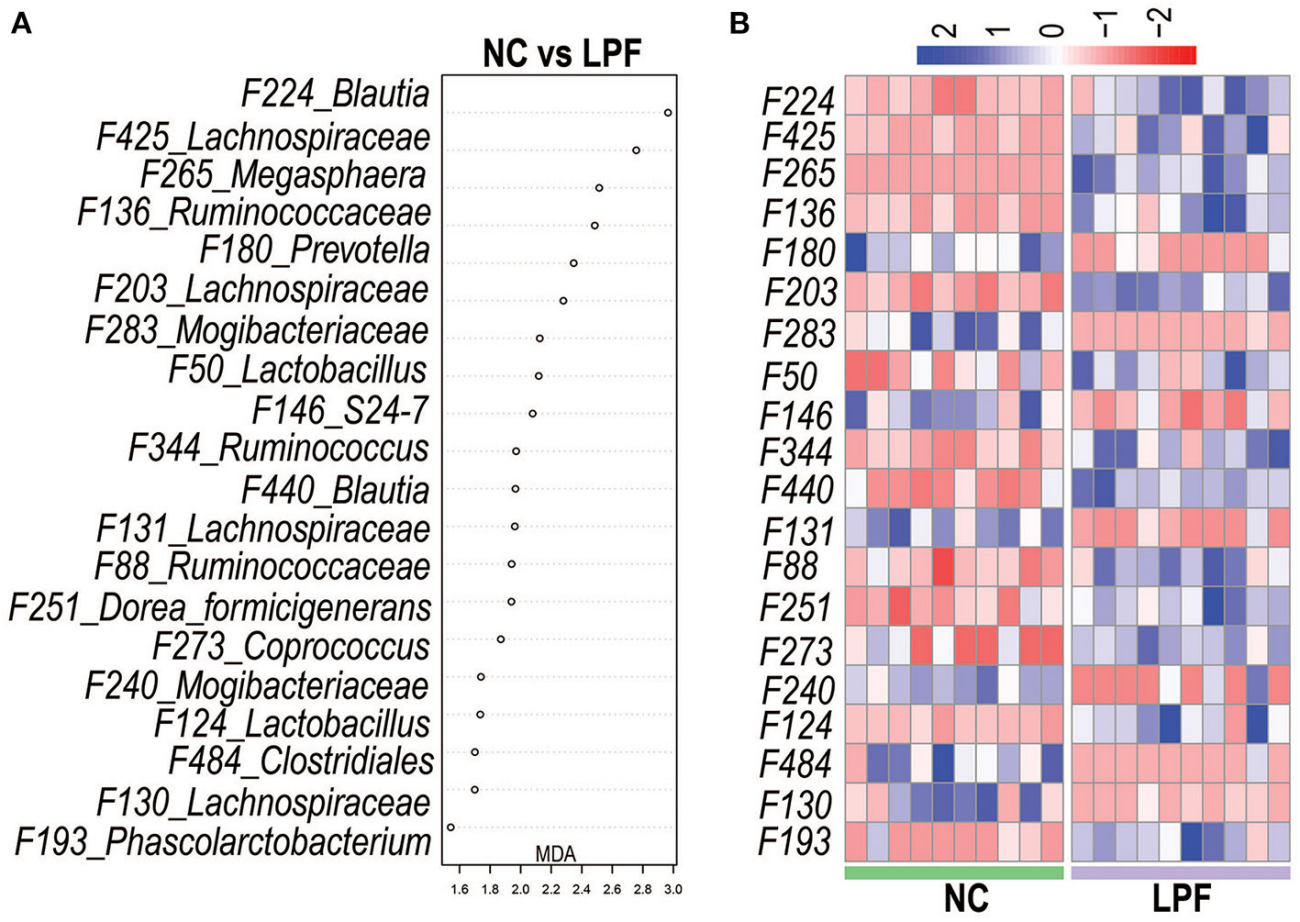
Gut microbiota signature of the pigs fed with LPF diet was determined by Random Forest. **(A)** Top 20 most predictive features that differentiate LPF-fed pigs from those fed with NC diet. **(B)** Heatmap shows the relative abundance of Features (log 10 transformed) selected by Random Forest. NC diet: basal diet, LPF diet: NC + 10% *Lactobacillus plantarum* and *Pediococcus acidilactici* co-fermented feed.

Microbial signatures that best differentiate the NC and LPF treatments were identified by random forest at the species level. The relative abundances of the top 500 bacterial features were included in the random forest model and the top 20 bacterial features that best predicted treatment are listed in Figure 6A. The relative abundances of these features for individual piglets are visualized on a heatmap (Figure 6B). Two members of *Lactobacillus* (Features #50 and #124) and *Blautia* (Features #224 and #440) were more abundant in the LPF group.

## Discussion

In the current study, the supplementation of 10% *Lactobacillus plantarum* and *Pediococcus acidilactici* co-fermented feed numerically increased the ADG, which is in accordance with previous reports that dietary fermented feed exhibited beneficial effects on swine production (15–18). However, several studies showed inconsistent results on the effects of fermented feed on growth performance. For example, Le et al. reported that *Lactobacillus reuteri* fermented wheat failed to improve the growth performance of weaned piglets (19). Liu et al. also observed that *Bacillus subtilis* fermented corn bran had no effects on ADFI and ADG of finishing pigs (20). The contradictory results could, at least in part, be explained by the different experimental animal ages and the different composition of fermented substrates, probiotic strains, as well as addition amount applied to the diet.

Weaning is the most stressful event in pig life, which could cause adverse impacts on gut health, such as villous atrophy, crypt hyperplasia, increased gut permeability, and intestinal inflammation (21). However, the SCFAs are considered beneficial to the gut and thus could help reduce weaning stress. A study conducted by Diao et al. demonstrated that SCFAs improved intestinal barrier function and reduced *E. coli* count in the ileal digesta in weaned piglets. Other studies confirmed that SCFAs help maintains gut barrier integrity (22, 23), which prohibits pathogens, toxins, or food proteins to pass into the blood. In addition, SCFAs have beneficial immune system effects in the intestinal mucosa (24, 25). Butyrate, a four-carbon short-chain fatty acid, is a primary energy source for intestinal epithelial cells and is anti-inflammatory (26). A study showed that butyrate improved the growth performance of weaning pigs fed diets containing 0.5% benzoic acid (27). Researchers also found that a mixture of SCFAs (propionic and formic) and capric acid significantly improved the growth performance of piglets (28). Overall, these findings suggested that SCFAs could reduce the detrimental effects of weaning stress and improve the growth performance of piglets. In the current study, the significantly increased acetate, butyrate, and total SCFAs may contribute to improved growth performance.

Consistent with previous studies (27, 29), our data showed that the Firmicutes and Bacteroidetes were the two dominant phyla and *Prevotella* was the most abundant genus in the gut microbiota of nursery piglets. The abundance of *Prevotella* might be linked with the diet style. Studies have shown that the relative abundance of *Prevotella* strikingly increased after the dietary transition from sow milk to corn/soybean meal-based diets (27). A human study also found that the relative abundance of *Prevotella* was associated with dietary habits and a high percentage of *Prevotella* could be a consequence of high fiber intake, improving metabolic energy absorption from consumed plant polysaccharides (30). These indicated that the gut microbiota coevolved with the diet.

LEfSe analysis was applied to identify the microbiota that was significantly changed by the LPF diet. Compared with the NC diet, LPF treatment significantly enriched SCFA-producing bacteria such as *Megasphaera, Roseburia, Faecalibacterium, Blautia, Selenomonas, Dialister, Acidaminococcus, Ruminococcus*, and *Bifidobacterium* (31–41). Some of those bacteria also have other beneficial functions, for example, *Megasphaera* could enhance large intestine functions and effectively prevent hyper-lactate accumulation-related diarrhea (42), *Roseburia* has benefits from immune modulation to inflammatory regulation (43), *Blautia* and *Faecalibacterium* had anti-inflammatory functions to help support gut health (44–46). In addition, this study indicated that the LPF diet selectively decreased the relative abundance of *Bacteroides*, an acetate producer (47). It may be that co-fermented feed additive prohibited the growth of *Bacteroides* directly or assisted the growth of antagonistic species against *Bacteroides* within the gastrointestinal tract. Taken together, our data indicated that the LPF diet improved the relative abundance of bacteria with potential probiotic properties.

In conclusion, this study demonstrated that the *Lactobacillus plantarum* and *Pediococcus acidilactici* co-fermented feed additive improved weaning pigs' growth performance, modulated gut microbiota diversity and composition, leading to enrichments of SCFA-producing bacteria.

## Data availability statement

The datasets presented in this study can be found in online repositories. The names of the repository/repositories and accession number(s) can be found at: https://www.ncbi.nlm.nih.gov/, https://www.ncbi.nlm.nih.gov/bioproject/PRJNA891080.

## Ethics statement

The animal study was reviewed and approved by Institute of Animal Husbandry and Veterinary Medicine of Hebei Province Animal Care and Use Committee.

## Author contributions

YY, XWe, and QZ conceived and designed this experiment. YY, GY, XM, XWa, GL, ZZ, and SZ collected rectal samples and growth performance data. XWe and YY analyzed the data. XWe, YY, and QZ drafted and revised the manuscript with input from GY, XM, ZZ, SZ, GL, and XWa. All authors read and approved the final manuscript.

## Supplementary material

Supplementary Figure S1 Alpha diversity for different dietary supplements was measured by **(A)** Shannon index, **(B)** Observed species, and **(C)** Chao1.

Supplementary Table S1 Composition and nutrient levels of basal diets (air-dry basis).

Supplementary Table S2 Composition of *Lactobacillus plantarum* and *Pediococcus acidilactici* co-fermented feed.

Supplementary Table S3 Effects of fermented feed additive on growth performances of nursery pigs.

Supplementary Table S4 Effects of fermented feed additive on serum chemistry of nursery pigs.

Supplementary Table S5 Effects of fermented feed additive on short chain fatty acid concentration in fecal samples.
