# Supplementary figures and images for: Effects of Lactobacillus plantarum and Pediococcus acidilactici co-fermented feed on growth performance and gut microbiota of nursery pigs
Source: Front Vet Sci. 2022 Dec 12;9:1076906. doi: 10.3389/fvets.2022.1076906 (PMC9792139; doi:10.3389/fvets.2022.1076906)

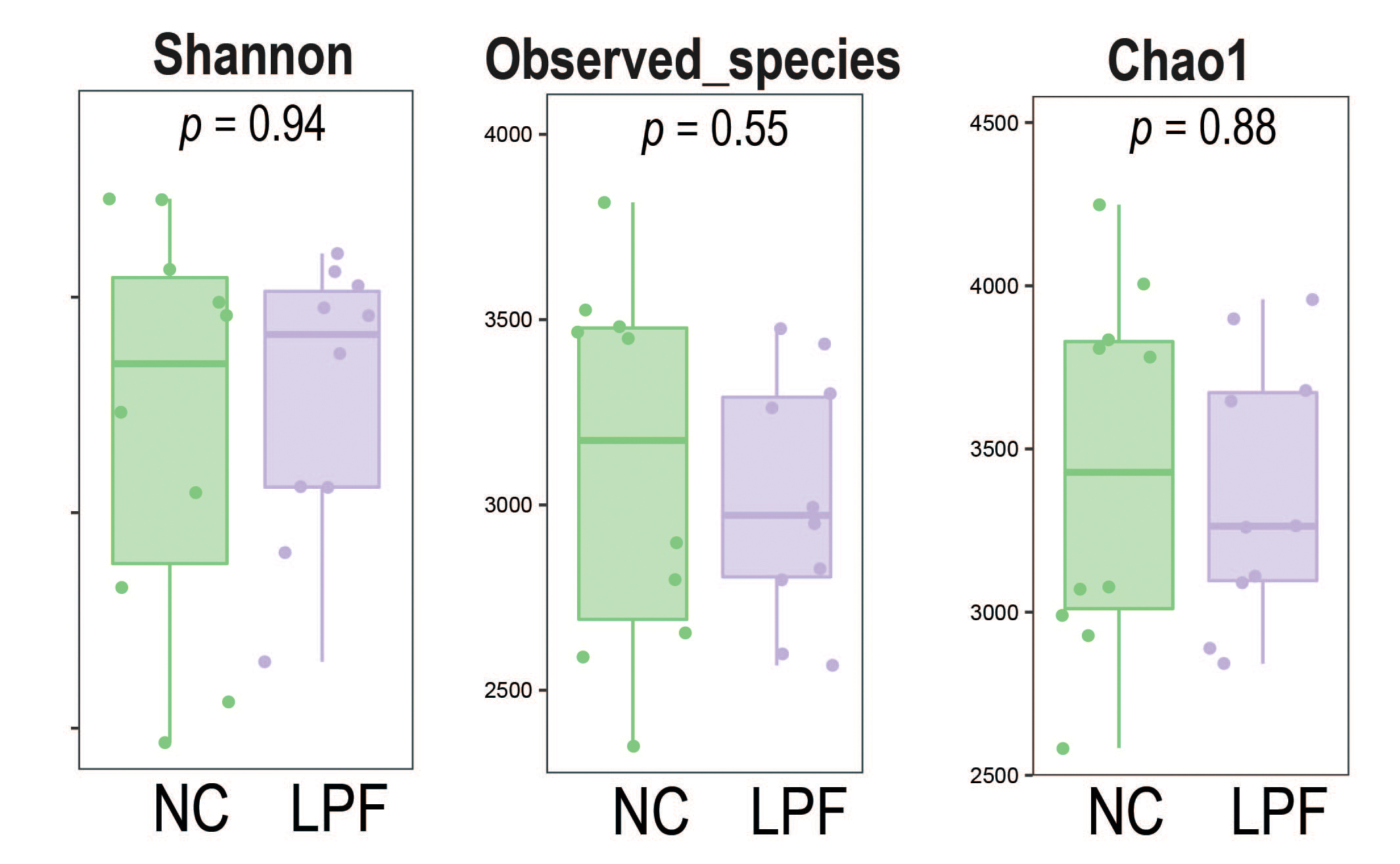

Supplement: Supplementary Figure S1 — Alpha diversity for different dietary supplements was measured by (A) Shannon index, (B) Observed species, and (C) Chao1. [file Image_1.jpg]
