# Supplementary material for: Effects of Lactobacillus plantarum and Pediococcus acidilactici co-fermented feed on growth performance and gut microbiota of nursery pigs
Source: Front Vet Sci. 2022 Dec 12;9:1076906. doi: 10.3389/fvets.2022.1076906 (PMC9792139; doi:10.3389/fvets.2022.1076906)
Supplement: Supplementary Table S1 — Composition and nutrient levels of basal diets (air-dry basis). [file Data_Sheet_1.docx]

Table S1: Composition and nutrient levels of basal diets (air-dry basis)

| Ingredients | Percentage |
| --- | --- |
| Corn | 63 |
| Soybean meal | 25 |
| Premix^1^ | 12 |
| Total | 100 |
| Nutrient levels^2^ |  |
| DE/(MJ/kg) | 12.86 |
| CP | 17.33 |
| Lys | 0.90 |
| Ca | 0.68 |
| TP | 0.54 |

^1^The premix provided the following per kg of diets: VA 5000 IU、VD 600 IU、VK_3_ 2.2 IU、VE 20 IU、VB_12_ 27.6 ug、VB_2_ 5.5 mg、D- pantothenic acid 14.8 mg、Nicotinic acid 30.3 mg、Choline 500mg、Cu 50mg、Fe 100mg、Zn 50mg、Mn 10 mg、I 0.85mg、Se 0.25mg；Ferrous fumarate 400mg/t

^2^Calculated value.

Table S2: Composition of *Lactobacillus plantarum* and *Pediococcus acidilactici*

co-fermented feed

| Ingredients, % | DM | CP | CF | Ash | Ca | TP |
| --- | --- | --- | --- | --- | --- | --- |
| Levels | 63.35 | 16.96 | 1.66 | 3.35 | 0.65 | 0.80 |

Note: pH for this co-fermented feed is 4.58.

Table S3: Effects of fermented feed additive on growth performances of nursery pigs.

|  | NC (*n*=16) | LPF (*n*=16) | SEM | *p*-Value |
| --- | --- | --- | --- | --- |
| Initial BW, kg | 14.58 | 14.73 | 0.93 | 0.878 |
| Final BW, kg | 33.31 | 34.63 | 1.85 | 0.484 |
| ADG, g | 604.24 | 641.94 | 43.40 | 0.392 |

Pigs were fed with experimental diets for 31 days. BW: body weight. ADG: average daily gain. NC diet: basal diet, LPF diet: NC + 10% *Lactobacillus plantarum* and *Pediococcus acidilactici* co-fermented feed.

Table S4: Effects of fermented feed additive on serum chemistry of nursery pigs.

|  | NC (*n*=5) | LPF (*n*=5) | SEM | *p*-Value |
| --- | --- | --- | --- | --- |
| TP, mg/mL | 54.24 | 74.00 | 4.21 | 0.002 |
| BUN, mg/mL | 0.27 | 0.33 | 0.04 | 0.196 |
| GPT, nmol/min/mL | 78.50 | 80.58 | 4.04 | 0.621 |
| GOT, nmol/min/mL | 55.10 | 54.55 | 2.89 | 0.856 |
| SOD, U/mL | 28.95 | 33.45 | 1.51 | 0.017 |

Pigs were fed with experimental diets for 31 days. NC diet: basal diet, LPF diet: NC + 10% *Lactobacillus plantarum* and *Pediococcus acidilactici* co-fermented feed.

Table S5: Effects of fermented feed additive on short chain fatty acid concentration in fecal samples

|  | NC (*n*=4) | LPF (*n*=4) | SEM | *p*-Value |
| --- | --- | --- | --- | --- |
| Acetate, μg/g | 276.84 | 528.35 | 80.53 | 0.020 |
| Propionate, μg/g | 177.62 | 219.80 | 23.34 | 0.121 |
| Isobutyrate, μg/g | 52.13 | 69.93 | 10.30 | 0.135 |
| Butyrate, μg/g | 210.30 | 293.39 | 25.46 | 0.017 |
| Isovalerate, μg/g | 67.35 | 85.97 | 13.93 | 0.230 |
| Valerate, μg/g | 139.51 | 177.40 | 29.98 | 0.253 |
| Total SCFAs | 923.75 | 1374.84 | 166.04 | 0.035 |

Pigs were fed with experimental diets for 31 days. NC diet: basal diet, LPF diet: NC + 10% *Lactobacillus plantarum* and *Pediococcus acidilactici* co-fermented feed.
